# Occult leydig cell tumour and androgen-receptor positive breast cancer in a woman with severe hyperandrogenism

**DOI:** 10.1186/1757-2215-6-43

**Published:** 2013-07-01

**Authors:** Giovanna Saraceno, Valeria Barresi, Francesco Trimarchi, Salvatore Cannavo

**Affiliations:** 1 Department of Clinical and Experimental Medicine, Via Consolare Valeria, Messina, 98125, Italy; 2 Department of Pathology, University of Messina, Messina, Italy

**Keywords:** Hyperandrogenism, Polycythemia, Ovary, Androgen-receptor, Breast

## Abstract

Leydig cell tumours represent more than 75% of all testosterone-secreting ovarian masses. These benign tumours are frequently occult or very small, but cause dramatic virilization. Chronic hyperandrogenism can also induce systemic complications, which increase morbidity and mortality risk. One of the most obvious effects of increased testosterone levels is polycythemia, a complication which induces dermatologic, osteoarticular and gastrointestinal manifestations and is associated with increased thrombotic risk. However, scientific literature reports few data concerning etiopathogenesis and management of polycythemia in patients with Leydig cell tumours. Moreover, no data are available about the effect of androgen excess on other concomitant tumours expressing androgen receptors. In this paper we report for the first time the case of a woman, with previous infertility, dramatic virilisation and chronic erythrocytosis, who was affected by an occult Leydig cell tumour and an androgen receptor positive breast cancer. This association gives us the opportunity to discuss the role of the steroid receptor expression of breast cancer in the presence of circulating androgen excess. Moreover, we demonstrate for the first time that treatment with flutamide (anti-androgen drug) is able to normalize blood cell count and haematocrit, before of achieving the definitive cure of hyperandrogenism by oophorectomy.

## Background

Severe female hyperandrogenism is an endocrine disorder characterized by serum testosterone levels in the masculine range. This is a very rare condition which is caused by adrenal or ovarian dysfunction, genetic or proliferative diseases. In the majority of cases hyperandrogenism occurs during the fertile adult life often due to a gonadal or adrenal virilising tumour. Hyperandrogenism can be extremely precocious when it is caused by congenital adrenal hyperplasia or more delayed when associated with ovarian hyperthecosis [1,2].

Androgen-secreting tumours account for <0.1% of all ovarian masses and comprise at least three categories: stromal luteoma, Leydig cell tumour and not otherwise specified steroid cell tumour [3,4]. Sometimes, they can be large and multifocal neoplasms, but in many cases these tumours are challenging to detect by non invasive imaging techniques, because they are small and unilateral [2,4,5]. The dramatic increase of testosterone levels induces typical phenotypic effects and, sometimes, systemic complications, especially due to erythropoiesis involvement [4,6-8]. Erythrocitosis is the result of direct bone marrow stimulation, red blood overproduction and induction of renal erythropoietin synthesis. However, despite this effect of testosterone is well-known, the association of androgen-secreting tumour and erythrocytosis has been reported at present only in a man with testicular Leydig cancer [6], in a woman with an ovarian steroid cell tumour [7], in another patient with an endometriod tumour [8], and in a woman with a large Leydig Cell Tumour [4]

Here, we present the case of a middle-age woman affected by an occult androgen secreting tumour of ovary causing erythrocytosis and by a breast cancer expressing only androgen receptors (AR) and, at lower extent, progesterone receptors (PR). The association of these two neoplasms has not been reported until now and we will speculate on the proliferative role of severe hyperandrogenism on clusters of AR positive (AR^+ve^) breast tumour cells.

## Case presentation

### History of patient

A 56-year old woman was referred to our Endocrine Unit because of severe virilisation. Since the age of 48, she complained of progressive, dramatic hirsutism, receding hairline, male-like muscular hypertrophy and voice deepening. She referred normal menses from the age of 12 to 45, when she became amenorrhoic. She reported spontaneous pregnancy at the age of 34, after multiple unsuccessful ovulation inductions. During the preceding seven years, bloodletting treatments were performed twice a month, because of severe polycythemia (RBC count: 5.95 million cells/μL; Haemoglobin: 14g/dl; haematocrit level >45%) without increased serum erythropoietin levels (18.8 mIU/ml, n.v. 3.7-31.5 - see Table 1). The occurrence of normal erythropoietin concentrations were in keeping with a similar observation previously reported by Yetkin et al. [4], suggesting that erythropoietin secretion could not be involved in hyperandrogenism-associated polycythemia. Three months before our evaluation the patient experienced a stroke. Since then she was on warfarin and levetiracetam treatment.

**Table 1:** Hormonal profile

**Hormone**	**Basal**	**After surgery (oophorectomy)**	**Normal range**
Total testosterone	**1148.0** ng/dl	7.16 ng/dl	6–82
Free testosterone	**22.3 **pg/ml	0.4 pg/ml	0.29–3.20
Bioavailable testosterone [CALCULATED]	**847** ng/dl	4.26ng/dl	
SHBG	18.9 nmol/L	16.8 nmol/L	20–85
Androstenedione	**3.10** ng/ml	1.5 ng/ml	0–2.83
DHEAS	107.5 ug/dl	85.81 ug/dl	35–430
17OHPG	2.80 ng/ml	0.4 ng/ml	
Cortisol	16.9 ug/dl		5–25
FSH	0.56 mIU/ml	17.34 mIU/ml	
LH	<0.1 mIU/ml	12.49 mIU/ml	
E2	77.76 pg/ml	12.2 pg/ml	
Pg	0.21 ng/ml	0.14 ng/ml	
5α DHT	**950** pg/ml		10–181
EPO	18.8 mIU/ml		3.7–31.5

Clinical examination showed facial plethora, BMI 32 kg/m^2^ (WHR: 0.98) and diastolic hypertension (BP: 130/100 mmHg). Ferriman-Gallwey evaluation (FG) scored 32. Gynaecological examination was negative, except for clitoromegaly and a huge solid, firm and irregular mass detected in the outer-lower quadrant of the left breast. For this reason she underwent lumpectomy, before endocrine investigation. Since histology revealed infiltrative ductal breast carcinoma, the patient underwent conventional radiotherapy (total dose: 45 Gy).

### Endocrine evaluation

Results of endocrine evaluation, performed after radiotherapy, are shown in Table 1. Free and total testosterone and dihydrotestosterone values were dramatically increased. Also serum 17-OH progesterone and androstenedione levels were slightly elevated. On the contrary, circulating cortisol and DHEAS concentrations were within the normal range. One-mg dexamethasone overnight administration induced appropriate suppression of cortisol and DHEAS levels (1.46 and 14.94 ug/dl respectively) and partially reduced 17-OH progesterone values (1.6 ng/ml). Serum LH and FSH levels were undetectable, while levels of other pituitary hormones were within the normal range. Ovarian tumour markers (CA 125, beta-HCG, CA 15.3, CEA) were also negative.

### Imaging

Trans-vaginal pelvic ultrasound and abdominal computed tomography (CT) were negative, showing normal ovarian size and morphology. Moreover, Positron Emission Tomography (PET), performed by Fluorine-18-Deoxyglucose and implemented by CT, was negative for abdominal or pelvic neoplasms. Other authors described previously usefulness of PET-CT in identification of small ovarian neoplasms [9,10].

### Clinical course

After starting the treatment for breast malignancy we advised oophorectomy supposing that the origin of increased testosterone levels might be placed in the ovary.

Since the patient refused surgery, she was treated by flutamide (500 mg/day, p. os) for six months. This therapy normalized blood cell count and haematocrit (RBC count: 4.84 million cells/μL; Haemoglobin: 12.4 g/dl; HCT 40%) in few weeks, allowing the withdrawal of bloodletting treatments (Table 1). Nevertheless, virilization features were slightly reduced (FG score = 26). On the other hand, treatment with Leuprorelin or other GnRH analogues, which is usually performed in patients with hyperthecosis or other gonadotropin-dependent hyperandrogenism, was inapplicable in our patient with undetectable FSH and LH levels [3,11,12]. Finally the patient accepted to undergo bilateral oophorectomy, which allowed the identification of a 8-mm ovarian tumour within the right ovary.

Seven days after oophorectomy, testosterone levels decreased dramatically and gonadotropin values reached menopausal range (Table 1). Three months later, hirsutism slightly improved (FG score = 21) but plethora disappeared. Moreover, hemochromocytometric parameters normalized completely (RBC count: 4.72 million cells/μL; Haemoglobin: 12.5 g/dl; HCT 38%) without any medication, and bloodletting therapy was no longer required.

### Pathology

**Figure 1:**
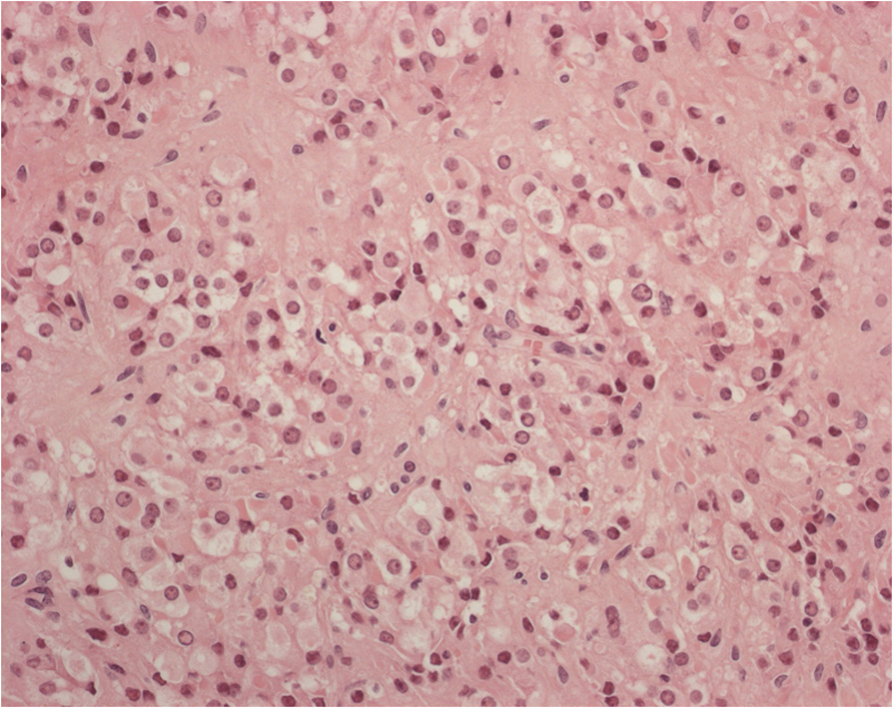
Leydig cell tumour composed by granular cells with eosinophilic cytoplasm and round nuclei (haematoxylin and eosin stain; original magnification, ×200).

Ovarian tumour was located at the hilum and composed by cells arranged in clusters, with granular cytoplasm, cytoplasmic inclusions and round nuclei (Figure 1). Pathology was indicative for the diagnosis of Leydig-cell tumour. Ki67 index was <1%.

**Figure 2:**
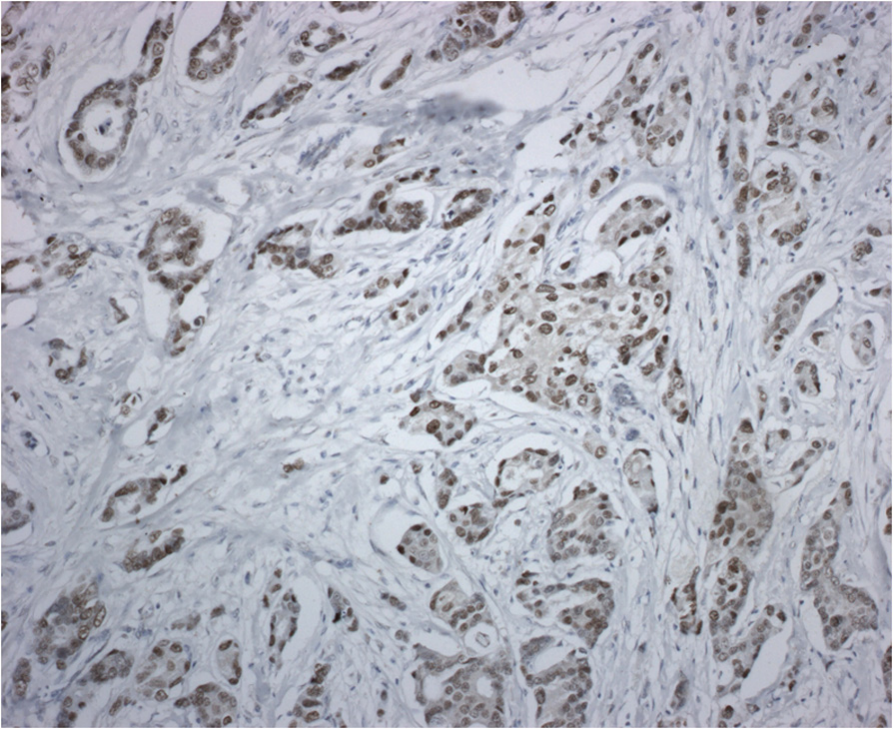
Nuclear expression of androgen receptor in the neoplastic cells of ductal carcinoma (androgen receptor stain; original magnification, ×200).

Breast tumour histological examination showed infiltrative ductal carcinoma, moderately differentiated (score 6 sec. Elston Ellis). Immunohistochemistry demonstrated that oestrogen receptors (ER) were negative (0%), but AR and PR were detected in 100% and 25% of tumour cells, respectively (Figure 2). HER2 status was negative (0%). Ki67 index was <10%. Some authors demonstrated that AR are expressed in 80% of invasive breast cancers, but only in 21% of all ER^-ve^ tumours [13]. On the contrary, most ER^+ve^ breast neoplasms are negative for AR [13]. In the experience of Micello et al. [14] AR is frequently expressed in ER^-ve^/PR^-ve^ breast cancer, in which expression of HER2 and AR is highly correlated. However, in our patient HER2-receptor expression was negative.

## Conclusions

In this paper we report for the first time the association of Leydig cell tumour and AR^+ve^ breast cancer in a woman with a long-term history of infertility, dramatic virilism and erythrocytosis.

The finding that the breast tumour did not express ER or HER2 suggests that cell proliferation can be stimulated by androgen excess, independently by circulating estrogens concentration if AR are expressed, and is in keeping with the *androgen-excess theory* recently proposed by Secreto and Zumoff [15]. These authors suggested that androgens play a central role in abnormal proliferation of breast tissue, and that hyperandrogenism, even mild or moderate, could be the underlying endocrine disorder that increases breast cancer risk. If the presence of androgen synthesising pathways can play a role on AR expression in breast tumour cells is therefore a big controversial in several study. Therefore, both estradiol and 5-dehydrotestosterone are considered to play important roles in the development of ductal invasive breast carcinoma as a result of the interactions of various enzymatic pathways, through an intracrine mechanism [16]. However until now has never been clarified the effect and the sequence of events resulting in the presence of “masculin range” of testosterone serum levels, precursor of DHT widely present in breast cancer tissues.

Our patient had a severe erythrocytosis requiring periodic bloodlettings. Recently Pelusi et al. described two similar cases of polycythaemia in their group of post-menopausal women with severe hyperandrogenism caused by Leydig cell tumours [17]. They provide an important perspective on the metabolic impact exerted by the woman’s exposure to high levels of circulating androgens. PCOS-associated hyperandrogenism has been associated with several metabolic abnormalities, such as impaired glucose tolerance, dyslipidemia, sistemic low grade inflammation, which cause a dramatic cerebro and cardiovascular risk [18]. The stroke occurred in our patient is in accordance with the increased vascular risk, esacerbated by the testosterone-induced stimulation of mature erythroid progenitors.

In patients with androgen-secreting ovarian tumours, treatment of choice for hyperandrogenism and its complications, i.e. polycythemia, is oophorectomy, but in this paper we demonstrated that antiandrogen drug flutamide is able to antagonize the effects of testosterone on bone marrow, normalizing hemochromocytometric parameters.

This drug is able to prevent the binding of circulating androgens to their receptors. The specific indication of flutamide concerns treatment of prostate cancer or benign prostatic hypertrophy. More recently it has been also introduced in the therapy of hyperandrogenism in young women. The drug has been well tollerated by our patient, but some side effects, as gastrointestinal disturbances, haemolytic anemia, muscle cramps, have been rarely reported in literature. Moreover, severe liver toxicity, sometimes fatal, has been demonstrated in few cases [19].

This drug, therefore, can be proposed in patients with increased surgical risk or who refuse oophorectomy.

## Consent

Written informed consent was obtained from the patient for publication of this Case Report and any accompanying images. A copy of the written consent is available for review by the Editor-in-Chief of this journal.

## Competing interests

The authors declare that they have no competing interests.

## Authors’ contributions

VB examined macroscopically and microscopically surgical specimen and performed the immunohistochemical staining of the tumours. GS, SC and FT conceived of the study, and helped to draft the manuscript. All authors read and approved the final manuscript.
